# Organprotektive Intensivtherapie und intensivmedizinische Maßnahmen bei Personen mit schwerer Hirnschädigung und unmittelbar bevorstehendem bzw. eingetretenem irreversiblen Hirnfunktionsausfall

**DOI:** 10.1007/s00101-025-01573-y

**Published:** 2025-08-25

**Authors:** Dominik Michalski, Lorenz Weidhase, Felix Pfeifer, Jan Sönke Englbrecht, Klaus Hahnenkamp, Svitlana Ziganshyna

**Affiliations:** 1 Klinik und Poliklinik für Neurologie, Universitätsklinikum Leipzig, Leipzig, Deutschland; 2 Interdisziplinäre Internistische Intensivmedizin, Universitätsklinikum Leipzig, Leipzig, Deutschland; 3 Deutsche Stiftung Organtransplantation, Region Ost, Leipzig, Deutschland; 4 Klinik für Anästhesiologie, operative Intensivmedizin und Schmerztherapie, Universitätsklinikum Münster, Münster, Deutschland; 5 Klinik für Anästhesiologie, Intensivmedizin, Notfallmedizin und Schmerzmedizin, Universitätsmedizin Greifswald, Greifswald, Deutschland; 6 Stabsstelle Transplantationsbeauftragte, Universitätsklinikum Leipzig, Philipp-Rosenthal-Str. 27b, 04103 Leipzig, Deutschland

**Keywords:** Organprotektive Intensivtherapie, Organprotektive intensivmedizinische Maßnahmen, Irreversibler Hirnfunktionsausfall, Hirntod, Organspende, Richtlinie Spendererkennung, Organ-protective intensive care, Organ-preserving critical care interventions, Irreversible loss of brain function, Brain death, Organ donation, Donor identification guidelines

## Abstract

**Hintergrund:**

Die im Jahr 2025 fortgeschriebene Richtlinie „Spendererkennung“ der Bundesärztekammer bekräftigt die Bedeutung der organprotektiven Intensivtherapie bzw. Anwendung intensivmedizinischer Maßnahmen zur Aufrechterhaltung der Organfunktionen bei unmittelbar bevorstehendem bzw. eingetretenem irreversiblen Hirnfunktionsausfall (IHA). Hierdurch soll die Möglichkeit einer Organspende bei Personen aufrechterhalten werden, wenn eine Bereitschaft zur Spende erklärt wurde oder diesbezüglich Unsicherheiten bestehen. Hinsichtlich der intensivmedizinischen Inhalte existiert in Deutschland bisher keine evidenzbasierte Leitlinie. Die vorliegende Arbeit soll daher eine Orientierungshilfe für die organprotektive Intensivtherapie bzw. Anwendung organfunktionserhaltender Maßnahmen bei Erwachsenen mit schwerer Hirnschädigung und unmittelbar bevorstehendem bzw. eingetretenem IHA bieten.

**Material und Methoden:**

Grundlage dieser Übersichtsarbeit waren englisch- und deutschsprachige Beiträge in entsprechenden Datenbanken (PubMed/Medline) sowie internationale Leitlinien und Handlungsempfehlungen.

**Ergebnisse:**

Innerhalb des Kontinuums vom unmittelbar bevorstehenden bis zum festgestellten IHA und der sich anschließenden Phase ergeben sich pathophysiologische Besonderheiten, die Implikationen für die organprotektive Intensivtherapie und entsprechende Maßnahmen zur Aufrechterhaltung der Organfunktionen haben. Diese beziehen sich v. a. auf die hämodynamische Situation, die Lungenfunktion, das endokrine System und infektiologische Komplikationen. Darüber hinaus existieren Maßnahmen, die auf den Schutz von Organen nach deren Entnahme abzielen. Auf der Basis von Kohorten‑/Register- bzw. wenigen randomisierten Studien konnten Maßnahmen in bestimmten Situationen eingegrenzt werden wie die i.v.-Gabe von Vasopressin und Glukokortikoiden sowie die konsequente lungenprotektive Beatmung mit Rekrutierungsmanövern, die mit einer Verbesserung der Hämodynamik und Lungenfunktion einherzugehen scheinen. Diesen Beobachtungen ist jedoch eine insgesamt geringe Evidenz gemeinsam, und Detailfragen, beispielsweise hinsichtlich des optimalen Zeitpunkts einer Vasopressin- bzw. Glukokortikoidgabe, sind ungeklärt. Darüber hinaus existierten anhaltend kontrovers diskutierte Maßnahmen wie die Substitution von Schilddrüsenhormonen, die niederschwellige Insulingabe und die Anwendung von Dopamin.

**Diskussion:**

Aus der vorhandenen Literatur lassen sich Empfehlungen für die Anwendung organprotektiver bzw. organfunktionserhaltender intensivmedizinischer Maßnahmen im zeitlichen Zusammengang mit dem IHA allenfalls bei Vorliegen bestimmter Kontextfaktoren wie einer instabilen hämodynamischen Situation ableiten. In der klinischen Praxis sollten daher allgemeine intensivmedizinische Standards zur Anwendung kommen. Notwendig sind Forschungsinitiativen, die sowohl spezifische intensivmedizinische Inhalte als auch Versorgungsaspekte berücksichtigen. Hierfür bieten sich randomisierte Studien ebenso wie die Weiterentwicklung des Deutschen Nationalen Transplantationsregisters an, auf deren Grundlage entsprechende Erkenntnisse generiert werden könnten.

## Abstract (en)

**Background:**

The 2025 revision of the donor identification guidelines by the German Medical Association reaffirms the importance of organ-protective intensive care or the application of intensive medical measures to maintain organ function in cases of imminent or established irreversible loss of brain function (brain death). These measures aim to preserve the possibility of organ donation in patients who have declared a willingness to donate or in whom the donation status remains uncertain. Currently, no evidence-based guidelines exist in Germany regarding the intensive care management in this context. This review aims to provide practical guidance on organ-protective intensive care and organ function-preserving strategies in adult patients with severe brain injury and imminent or confirmed brain death.

**Material and methods:**

This review is based on relevant English and German language publications retrieved from databases (PubMed/Medline) as well as international guidelines and expert recommendations for action.

**Results:**

Across the continuum from imminent to confirmed brain death and the subsequent phase, various pathophysiological changes occur that have implications for organ-protective intensive care and the maintenance of organ function. These include disturbances in hemodynamics, lung function, the endocrine system and infectious complications. Additionally, some measures focus on organ protection after retrieval. Based on cohort and registry studies and few randomized trials, interventions such as intravenous administration of vasopressin and glucocorticoids as well as consistent lung-protective ventilation including recruitment maneuvers, appear to improve hemodynamics and lung function in certain situations; however, the overall evidence remains limited and key aspects, such as the optimal timing for vasopressin or glucocorticoid administration, remain unresolved. Other interventions, such as thyroid hormone substitution, low-threshold insulin administration, and the use of dopamine, continue to be controversially discussed.

**Discussion:**

From the current literature recommendations can be derived regarding the use of organ-protective intensive care or organ function-preserving strategies in the context of brain death, mainly under specific conditions such as hemodynamic instability. In the clinical practice, general standards of intensive care should therefore be applied. Further research initiatives are urgently needed to address both specific medical interventions and healthcare supply aspects. This could be achieved through randomized trials and the further development of the German National Transplantation Registry, which would provide a foundation for generating robust clinical insights.

## Hintergrund

Die Zahl der in Deutschland realisierten Organspenden und damit auch der gespendeten Organe hatte in den 2010er-Jahren einen deutlichen Rückgang erfahren und 2017 einen Tiefpunkt erreicht [1, 2]. Diese Entwicklung kontrastierte mit der Beobachtung, dass die Zahl der Personen, die potenziell für eine Organspende infrage kommen, in einem vergleichbaren Zeitraum um ca. 14 % zunahm [3]. Im Jahr 2023 war mit 965 realisierten Spenden und 2877 gespendeten Organen eine graduelle Zunahme erkennbar [2], die jedoch bei Weitem nicht zu einer Deckung des Bedarfs an Organen führt.

Zu den Initiativen, die auf eine zahlenmäßige Erhöhung der Organspenden abzielen, gehört die im Jahr 2020 veröffentlichte Richtlinie „Spendererkennung“ der Bundesärztekammer [4]. Diese soll sicherstellen, dass für eine Organspende infrage kommende Personen in den behandelnden Krankenhäusern zuverlässig erkannt werden. Gemeint sind Personen mit schwerer primärer oder sekundärer Hirnschädigung insbesondere infolge einer Hirnmassenblutung, eines raumfordernden Hirninfarkts, eines Schädel-Hirn-Traumas oder einer hypoxisch-ischämischen Enzephalopathie, bei denen ein irreversibler Hirnfunktionsausfall (IHA) unmittelbar bevorsteht oder richtliniengemäß bereits festgestellt wurde [5]. Die Richtlinie „Spendererkennung“ wurde jüngst fortgeschrieben und bekräftigt in diesem Zusammenhang die Notwendigkeit der Eingrenzung eines zuvor geäußerten oder mutmaßlichen Willens der Betroffenen für oder gegen eine Organspende, da dies Einfluss auf die individuelle Behandlungsplanung hat. Bei bestehenden Unsicherheiten hinsichtlich der Spendebereitschaft sollen intensivmedizinische Maßnahmen eingeleitet bzw. fortgeführt werden, um die Möglichkeit einer Organspende aufrechtzuhalten [6].

Angesichts des bestehenden Bedarfs an Spenden kommt der Qualität der organprotektiven Intensivtherapie vor der Feststellung des IHA und der im Nachgang notwendig werdenden intensivmedizinischen Maßnahmen, welche auf eine Aufrechterhaltung der Organfunktionen abzielen, eine große Bedeutung zu. Hinsichtlich deren inhaltlicher Ausgestaltung existieren in Deutschland anders als beispielsweise in Kanada [7], Irland [8], Indien [9] und den USA [10] keine konkreten Handlungsempfehlungen bzw. Leitlinien, die sich im Sinne einer Evidenzsynthese aus der wissenschaftlichen Literatur ableiten. Zudem fokussieren die verfügbaren Übersichtsarbeiten überwiegend auf den Zeitpunkt des Eintritts des IHA bzw. die nachfolgende Phase bis zur Organentnahme [11–14].

Die vorliegende Arbeit stellt daher eine Orientierungshilfe für die organprotektive Intensivtherapie bzw. die Anwendung intensivmedizinischer Maßnahmen bei Erwachsenen mit schwerer Hirnschädigung und unmittelbar bevorstehendem bzw. eingetretenem IHA dar. Hierfür werden die in den vergangenen Jahren international diskutierten, organprotektiven und auf die Aufrechterhaltung der Organfunktionen ausgerichteten, intensivmedizinischen Aspekte anhand von Studien aufgearbeitet und Anregungen für Forschungsinitiativen gegeben.

## Methode

Für diese Übersichtsarbeit erfolgte eine Literatursuche unter Verwendung frei zugängiger Datenbanken (PubMed/Medline), begrenzt auf englisch- und deutschsprachige Beiträge. Um v. a. neue Erkenntnisse abzubilden, wurden bevorzugt die in den vergangenen 10 Jahren (ab 2015) veröffentlichten Arbeiten berücksichtigt, jedoch themenspezifisch auch frühere Arbeiten herangezogen. Darüber hinaus gingen frei zugängige, internationale Leitlinien und Handlungsempfehlungen sowie eigene Erfahrungen der Verfassenden ein.

## Ergebnisse

### Vorbemerkungen

Im zeitlichen Zusammenhang mit dem IHA ergeben sich zahlreiche pathophysiologische Besonderheiten wie beispielsweise der oft auftretende (zentrale) Diabetes insipidus [15], die unmittelbar Implikationen im intensivmedizinischen Verlauf haben [12]. Zum Zeitpunkt des noch nicht eingetretenen IHA besteht die Herausforderung, diese Aspekte zu antizipieren und im Falle des Auftretens frühestmöglich zu adressieren, gleichzeitig aber eine prophylaktische Therapie insbesondere dann zu vermeiden, wenn mit dieser negative Effekte verbunden sein könnten. Ein ethisches Dilemma entsteht dabei nicht zwangsläufig, da Maßnahmen, die auf eine optimale Organfunktion bei noch nicht eingetretenem IHA abzielen, als Bestandteil einer hochwertigen intensivmedizinischen Versorgung angesehen werden können, solange keine grundsätzliche Ablehnung dieser besteht. Kontrovers können Maßnahmen, für die positive Effekte hinsichtlich der Organfunktion im Vorfeld des IHA nicht belegt sind, aber sich mögliche Vorteile im Nachgang einer Transplantation abzeichnen und somit ein Nutzen ausschließlich für die Organempfangenden anzunehmen ist, diskutiert werden. Inwiefern diese Maßnahmen unter der Bedingung einer vorhandenen Spendebereitschaft bereits vor der Feststellung des IHA zur Anwendung kommen können oder gar sollen, bedarf vermutlich einer umfangreichen medizinethischen Debatte, die nicht Bestandteil der vorliegenden Arbeit sein soll. Zumindest für erweiterte intensivmedizinische Maßnahmen (mechanische Reanimation etc.), für die sich innerhalb des Kontinuums von der organprotektiven Intensivtherapie bei bevorstehendem IHA über dessen Feststellung bis zur sich anschließenden Phase mit letztlich erfolgender Organentnahme ebenfalls unterschiedliche ethische Perspektiven ergeben können, existiert ein Positionspapier der Deutschen Interdisziplinären Vereinigung für Intensiv- und Notfallmedizin [16], welches als Entscheidungshilfe herangezogen werden kann.

Als praxisnahe Orientierungshilfe fokussiert die vorliegende Arbeit auf die Themenbereiche Hämodynamik, Lungenfunktion, endokrine Störungen sowie Inflammation und Infektion unter Berücksichtigung pathophysiologischer Besonderheiten, die im zeitlichen Zusammenhang mit dem IHA bestehen. Auszugsweise sollen darüber hinaus Ansätze, die auf den Schutz von Organen nach deren Entnahme abzielen, berücksichtigt werden. Wenngleich Unsicherheiten verbleiben, erlaubt die in den vergangenen Jahren verfügbar gewordene Literatur in Verbindung mit allgemeinen intensivmedizinischen Prinzipien Hinweise für die Umsetzung organprotektiver bzw. organfunktionserhaltender Maßnahmen, die in Tab. 1 zusammengefasst sind. Hinsichtlich weiterer intensivmedizinischer Inhalte wie speziellen Aspekten der Beatmung, der Temperaturkontrolle, der Ernährung, der Volumentherapie, des Gerinnungsmanagements sowie der Prophylaxe venöser Thromboembolien und gastrointestinaler Ulzera ergibt sich keine Rationale, von allgemeinen Empfehlungen abzuweichen [17–19].

**Tab. 1:** Hinweise für die praktische Umsetzung organprotektiver bzw. organfunktionserhaltender intensivmedizinischer Maßnahmen (in Anlehnung an: [7–12, 17, 29, 72, 73, 92–95])

	Ziele	Abweichung/Interventionsmöglichkeit	Anmerkungen
*Hämodynamik*	– Euvolämie – Mittlerer arterieller Druck 65–90 mm Hg – Normales Serumlaktat (≤ 2 mmol/l) – Normale Mikrozirkulation: – Normale Rekapillarisierungszeit (< 2–3 s) – Keine Marmorierung – Ausreichend warme Peripherie – Ausreichende Diurese (1–3 ml ⋅ kg^-1^ ⋅ h^-1^)	*Volumenmangel:* – Gabe von balancierten Vollelektrolytlösungen	Für Beurteilung des Volumenstatus Nutzung funktioneller Tests (z. B. „Passive-leg-raise“-Manöver oder „rapid fluid challenge“) bzw. dynamischer Vorlastparameter (z. B. SVV, PPV)
		*Hypervolämie:* – Diuretika (Furosemid, Torasemid; ggf. Thiaziddiuretika bei erhöhtem Natrium), ggf. Nierenersatztherapie	
		*Peripheres Kreislaufversagen:* – Vasopressin (z. B. 0,04 U/min) – Noradrenalin (bedarfsabhängige Dosierung)	Frühzeitiger Einsatz von Vasopressin kann den Kreislauf stabilisieren und zur gezielten Behandlung des Diabetes insipidus beitragen
		*Unzureichendes Herzzeitvolumen und reduzierte linksventrikuläre Ejektionsfraktion:* – Gabe von Dobutamin erwägen (< 10 μg ⋅ kg^-1^ ⋅ min^-1^)	Bei neuer linksventrikulärer Dysfunktion kritisches Hinterfragen des Einsatzes positiv- inotroper Substanzen analog zur Takotsubo-Kardiomyopathie
		*Hypertonie:* – Urapidil (10–50 mg i.v. als Bolus, nachfolgend 2–15 mg/h) – Nitroprussid-Natrium (0,5–5 μg ⋅ kg^-1^ ⋅ min^-1^) oder Nitroglyzerin (0,5–8 μg ⋅ kg^-1^ ⋅ min^-1^)	
*Lungenfunktion*	– Periphere Sauerstoffsättigung (SaO_2_): 92–96 % – Arterieller Sauerstoffpartialdruck (PaO_2_): 70–90 mm Hg – pH 7,35–7,45 – Horovitz-Index (PaO_2_/FiO_2_) > 300	*Allgemein:* Lungenprotektive kontrollierte Beatmung mit den Charakteristika: – Tidalvolumen: 6–8 ml/kg Idealgewicht – Positiver endexspiratorischer Druck (PEEP): ≥ 8 cm H_2_O – Plateaudruck ≤ 30 cm H_2_O – Driving Pressure ≤ 14 cm H_2_O	
		*Oxygenierungsstörung mit Horovitz-Index <* *300:* – Optimierung des PEEP-Niveaus – Rekrutierungsmanöver erwägen – Bauchlagerung erwägen – Restriktives Volumenmanagement	Bei Horovitz-Index < 300 und Eignung zur Lungenspende Erwägung von Rekrutierungsmanövern (z. B. Erhöhung des PEEP auf 15 cm H_2_O für 2 h) bzw. nach kritischer Nutzen-Risiko-Abwägung auch Bauchlagerung (≥ 12 h)
		*Vor allem bei Sekretlast und Atelektasen:* – Bronchoskopie	Bei Eignung zur Lungenspende initial Bronchoskopie zur Beurteilung der Anatomie und mikrobiologischen Diagnostik; weitere Bronchoskopien in Abhängigkeit von Sekretlast, Atelektasen und Oxygenierung
*Endokrines System und Homöostase*	– Blutzucker: 6–10 mmol/l (< 180 mg/dl) – Kalium: 3,5–5 mmol/l – Natrium: 135–145 mmol/l – Normothermie: > 35 °C – Hb-Wert: ≥ 4,3 mmol/l (≥ 6,9 g/dl)	*Hyperglykämie:* – Bedarfsadaptiert Insulingabe	Insulingabe dient der Vermeidung relevanter Hyperglykämien, keine Vorteile einer niederschwelligen Insulingabe
		*Diabetes insipidus:* – Gabe von Desmopressin (1–4 μg i.v. 1–2 × täglich) bei manifestem zentralen Diabetes insipidus	Engmaschige Kontrollen der Serum-Natrium- und Urin-Natrium-Konzentration bzw. -osmolalität sowie der Diurese
		*Schilddrüsenhormonsubstitution:* – Keine generelle Empfehlung	Erwägung im Einzelfall bei hämodynamischer Instabilität und eingeschränkter linksventrikulärer Funktion
		*Hyper‑/Hypothermie:* – Vermeidung von Wärmeverlusten – Ggf. aktive Anwärmung (z. B. Wärmedecken)	
		*Anämie:* – Transfusion von Erythrozytenkonzentraten für Aufrechterhaltung Hb > 4,3 mmol/l (> 6,9 g/dl)	Leukozytendepletierte, gefilterte Erythrozytenkonzentrate, Transfusion von Blutprodukten, wenn möglich, nach Abnahme der Blutproben für HLA-Typisierung und virologische Untersuchungen
*Inflammation und Infektion*	– Ggf. Modulation der systemischen Immunreaktion	*Glukokortikoidsubstitution:* – Methylprednisolon (Bolus 250 mg i.v., anschließend 100 mg/h) – Hydrocortison (Bolus 100 mg i.v., anschließend 12,5 mg/h bzw. 0,18 µg ⋅ kg^-1^ ⋅ h^-1^)	Steroide können den Kreislauf stabilisieren und die Transplantatfunktion verbessern, Unsicherheiten existieren hinsichtlich der optimalen Substanz und Dosierung, daher Substitution gemäß intensivmedizinisch etablierten Schemata
	– Detektion und Behandlung von Infektionen	*Inflammationsparameter:* – Keine Empfehlung für alleinige Nutzung	Inflammationsparameter (CRP, PCT, IL-6) alleinig zur Diagnosestellung einer Infektion ungeeignet
		*Mikrobiologisches Monitoring:* – Insbesondere bei Fieber/V. a. Infektion Abnahme von Blutkulturen, respiratorischen Sekreten, Urin und ggf. weiterem Material	
		*Antibiotikatherapie:* – Kalkulierte antiinfektive Behandlung bei begründetem Verdacht auf Infektion	Keine prophylaktische Antibiotikagabe, jedoch bei Hinweisen auf eine pulmonale (Konversion des Bronchialsekrets, neue Infiltrate) oder extrapulmonale Infektion frühzeitig Beginn Antibiotikatherapie
*Organprotektion nach IHA-Feststellung*		*Dopamingabe:* – Keine generelle Empfehlung	Unsichere Datenlage

*SVV* stroke volume variation, *PPV* pulse pressure variation

### Hämodynamik und neu diagnostizierte Herzinsuffizienz

Die hämodynamische Instabilität ist eine der offensichtlichsten Herausforderungen während der organprotektiven Intensivtherapie bzw. Anwendung organfunktionserhaltender Maßnahmen. Infolge einer schweren Hirnschädigung mit ggf. bereits vorliegender zerebraler Perfusionseinschränkung kommt es zur Sympathikusaktivierung mit Anstieg des arteriellen Blutdrucks. Im weiteren Verlauf geht der Abfall der zuvor stark erhöhten Katecholaminlevel häufig mit einer Vasoplegie einher, wodurch ein Abfall des Blutdrucks in den Vordergrund rückt, was zumeist die differenzierte Volumen- und Katecholamintherapie, v. a. unter Einsatz von Vasopressoren, erforderlich macht [20–23].

Die beim Diabetes insipidus verminderte hypophysäre Freisetzung des antidiuretischen Hormons (ADH, *Vasopressin*) hat eine Polyurie und konsekutiv eine Hypovolämie mit hämodynamischer Instabilität zur Folge [23]. Die Gabe von Vasopressin bzw. des synthetischen Analogons Desmopressin dient daher der Behandlung des Diabetes insipidus mit dem übergeordneten Ziel der hämodynamischen Stabilisierung sowie Sicherstellung der Homöostase des Wasser- und Elektrolythaushaltes. Diskutiert wird die frühzeitige und teils bereits präventive Gabe von Vasopressin in niedriger Dosierung. Vorteile könnten sich durch einen reduzierten Bedarf an vasokonstriktorischen Katecholaminen und die Beeinflussung von inflammatorischen Prozessen ergeben, was zu verbesserten Organfunktionen im intensivmedizinischen Verlauf und nach einer Transplantation führen kann [24]. Auf der Basis kleiner Kohortenstudien aus den USA und Großbritannien finden sich Hinweise darauf, dass eine z. T. schon vor der Diagnose eines Diabetes insipidus initiierte, niedrigdosierte und kontinuierliche Gabe von Vasopressin (0,04–0,1 U/min bzw. 300 μU/kgKG und min) mit einer *verbesserten Hämodynamik und einem geringeren Katecholaminbedarf* einhergeht [25, 26]. Zwei umfangreiche Registerstudien unter Nutzung des US-amerikanischen Organ Procurement and Transplantation Network (OPTN) mit 10.431 bzw. 12.322 Fällen lieferten Hinweise auf einen positiven Einfluss der Vasopressingabe auf die Organfunktionen. Wenngleich der Behandlungszeitpunkt nicht spezifiziert wurde, war die Anwendung von Vasopressin mit einer höheren Anzahl an erfolgreich vermittelten Organen sowie einer geringeren Rate an Organablehnungen infolge einer schlechten Funktion assoziiert [27, 28]. Neben einem positiven Effekt auf die hämodynamische Situation und damit die Funktion einzelner Organe wird für den Einsatz von Vasopressin bzw. seiner Analoga während der organprotektiven Therapie auch ein vorteilhafter Effekt in Bezug auf das Transplantationsergebnis diskutiert [24]. Aus den vorhandenen Studien lassen sich jedoch noch keine Empfehlungen hinsichtlich des optimalen Zeitpunkts und der Dosis einer frühzeitigen oder sogar präventiven Gabe von Vasopressin ableiten. Wenngleich internationale Behandlungsempfehlungen Vasopressin für das hämodynamische Management im Kontext des IHA bereits einschließen (z. B. [7, 10]), kommt im deutschsprachigen Raum in erster Linie *Desmopressin,* vorrangig zur Behandlung des Diabetes insipidus, zum Einsatz. Der Zeitpunkt der Anwendung orientiert sich dabei in der Regel an der Diagnosestellung des Diabetes insipidus, wofür als Merkmale eine Polyurie (Diurese > 5 ml/ kgKG und h), ein Anstieg der Serum-Natrium-Konzentration/-Osmolalität sowie eine Reduktion der Urin-Natrium-Konzentration/-Osmolalität mit einem spezifischen Uringewicht von weniger als 1005 mg/l herangezogen werden [12, 29].

Im zeitlichen Kontext des IHA kann bei Personen ohne vorbestehende Herzerkrankung eine *linksventrikuläre Dysfunktion* auftreten. Ursächlich wird eine Stresskardiomyopathie durch erhöhte Katecholaminlevel angenommen; diese geht mit einer eingeschränkten Funktion des Myokards einher und wird echokardiographisch in Form von Hypokinesien sichtbar [30]. Untersuchungen auf der Basis einer US-amerikanischen prospektiven Kohortenstudie mit 3794 Fällen deuten darauf hin, dass eine relevant eingeschränkte linksventrikuläre Funktion (Ejektionsfraktion < 50 %) in bis zu 13 % der Personen mit einem IHA vorkommt. In 58 % dieser Fälle lieferte eine zweite echokardiographische Untersuchung mit einer Latenz von ca. 24 h eine Ejektionsfraktion ≥ 50 %, sodass sich Hinweise auf eine Reversibilität fanden [31]. Diskutiert wird in diesem Zusammenhang die Bedeutung einer linksventrikulären Dysfunktion für die *Transplantierbarkeit des Herzens* und den anschließenden Langzeitverlauf. Basierend auf der US-amerikanischen Datenbank United Network for Organ Sharing (UNOS) mit 30.993 Fällen zeigte sich bei 0,4 % der Spendenden eine linksventrikuläre Ejektionsfraktion < 40 % und in 2,0 % der Fälle von 40–50 %, wohingegen bei 97,6 % der Untersuchten keine relevante Einschränkung vorlag. Die Rate an Transplantatversagen bzw. einer Abstoßung des transplantierten Herzens unterschied sich dabei innerhalb eines Jahres nicht zwischen den Gruppen mit reduzierter und normaler linksventrikulärer Ejektionsfraktion. Ebenso unterschied sich die Überlebensrate der Organempfangenden nach einem Jahr mit 84,6 gegenüber 91,3 % nicht signifikant [32]. Auf echokardiographische Muster und Prädiktoren der linksventrikulären Dysfunktion fokussierte eine schwedische Studie mit 641 potenziell Spendenden, bei denen eine Ejektionsfraktion < 50 % bzw. regionale Hypokinesien in 24 % der Fälle vorlagen. Überwiegend handelte es sich dabei um regionale Wandbewegungsstörungen (18 %) und in geringerer Zahl um globale Dysfunktionen (5 %). Ein *Tako-Tsubo-Kardiomyopathie-typisches Bild* war bei 41 % der auffälligen echokardiographischen Befunde zu erheben. Dabei waren ein jüngeres Alter, eine psychiatrische Erkrankung, ein zerebrales Ödem als Ursache des IHA, ein Suizid, eine Intoxikation und ein Herzstillstand mit einer linksventrikulären Dysfunktion assoziiert. Gespendete Herzen mit initial vorhandener linksventrikulärer Dysfunktion zeigten in der echokardiographischen Kontrolle nach der Transplantation eine signifikant verbesserte Ejektionsfraktion von im Mittel 47 auf 55 %. Im Langzeitverlauf bis zu 10 Jahren unterschied sich die Sterblichkeit bzw. Rate an Retransplantationen nicht zwischen Personen mit transplantierten Herzen, die initial eine linksventrikuläre Dysfunktion aufwiesen bzw. dieses Merkmal nicht besaßen [33].

Bei der Bewertung der vorgenannten Studien ist eine mögliche Verzerrung zu berücksichtigen, da lediglich Herzen, die transplantiert worden sind, Berücksichtigung fanden. Informationen bezüglich der nichttransplantierten Herzen mit linksventrikulärer Dysfunktion liegen naturgemäß nicht vor. Dennoch zeigen die gemachten Beobachtungen, dass sich bei einem relevanten Teil der potenziell Spendenden ohne vorbestehende Herzerkrankung eine *unerwartet auftretende linksventrikuläre Dysfunktion bessern kann*, woraus sich Implikationen für organprotektive intensivmedizinische Maßnahmen und auch die Transplantierbarkeit ergeben können. Dabei ist gänzlich offen, ob spezifische organprotektive Maßnahmen mit dem Ziel einer raschen Steigerung der linksventrikulären Ejektionsfraktion, wie in einigen Arbeiten empfohlen [8], sinnvoll sind. Denkbar wäre speziell bei der Tako-Tsubo-Kardiomyopathie auch ein ungünstiger Effekt durch positiv-inotrop wirkende Substanzen.

### Lungenfunktion

Erhöhte Katecholaminlevel, wie sie im Rahmen schwerer zerebraler Schädigungen vorliegen, wirken sich nachteilig auf die Lungenfunktion aus, u. a. durch eine pulmonale Vasokonstriktion mit erhöhtem Druck innerhalb der Kapillaren und resultierender endothelialer Schädigung mit dem Risiko der Ödembildung. Hinzu kommen Faktoren wie aspirations- bzw. ventilatorassoziierte Pneumonien und evtl. Schäden durch vorangegangene Traumata [20, 34]. Die bestmögliche Aufrechterhaltung der Lungenfunktion stellt einen wichtigen Bestandteil der organprotektiven Therapie dar, einerseits mit dem Ziel der suffizienten Oxygenierung und damit Funktionssicherung anderer Organe, aber auch der Transplantierbarkeit der Lunge selbst. Für die Bewertung wird oft der Horovitz-Index (p_a_O_2_/F_I_O_2_) als globales Maß für die Lungenfunktion herangezogen, wobei Werte > 300 für eine suffiziente Funktion sprechen. Herangezogen werden auch radiologische Merkmale sowie eine bronchoskopische Beurteilung mit bestenfalls nichtvorhandenen Infiltraten und fehlendem purulenten Sekret oder Zeichen der Aspiration [35].

Zu den Maßnahmen, die auf eine Verbesserung der Lungenfunktion abzielen und im Zusammenhang mit dem IHA in Studien untersucht wurden, gehören die konsequente lungenprotektive Beatmung, die Rekrutierung von Alveolen durch eine Bauchlagerung und spezielle Rekrutierungsmanöver sowie Bronchoskopien, aber auch die Vermeidung einer Hypervolämie. Eine multizentrische randomisierte Studie unter Einbeziehung von 12 europäischen Intensivstationen untersuchte eine strikte *lungenprotektive Beatmung* gegenüber einem weniger restriktiven Vorgehen bei 118 potenziell Lungenspenden. In beiden Behandlungsgruppen wurde die Atemfrequenz derart angepasst, dass ein p_a_CO_2_ von 40–45 mm Hg resultierte und die inspiratorische Sauerstoffkonzentration (F_I_O_2_) so gewählt, dass ein p_a_O_2_ von mindestens 90 mm Hg aufrechterhalten werden konnte. Die lungenprotektive Strategie umfasste darüber hinaus die Anwendung eines positiven endexspiratorischen Drucks (PEEP) von 8–10 cm H_2_O, Tidalvolumina von 6–8 ml/kgKG (bezogen auf das ideale Körpergewicht) und eine Vermeidung der Diskonnektion während des Apnoetests. Infolge dieser Maßnahmen erfüllten 95 % der Lungen die Kriterien für eine Spende. Das konventionelle Beatmungskonzept umfasste einen PEEP von 3–5 cm H_2_O, Tidalvolumina von 10–12 ml/kgKG und eine Diskonnektion während des Apnoetests, worunter lediglich 54 % der Lungen für eine Spende infrage kamen. Diese Beobachtung führte zu einem vorzeitigen Abbruch der Studie, wenngleich sich die Sterblichkeit der Organempfangenden innerhalb von 6 Monaten nach der Transplantation zwischen den Beatmungsstrategien nicht unterschied [36]. Zu vergleichbaren Ergebnissen kamen eine in Frankreich durchgeführte Kohortenstudie mit 1626 potenziell Lungenspendenden und eine auf dem US-amerikanischen OPTN-Datensatz beruhende Registerstudie mit 6052 Fällen [37, 38].

Den Effekt einer *Bauchlagerung* im Kontext des IHA untersuchte eine Kohortenstudie mit 134 potenziell spendenden Personen, die Zeichen einer insuffizienten Oxygenierung (Horovitz-Index < 300) sowie Atelektasen aufwiesen. Eine Bauchlagerung über mindestens 12 h ging mit einem signifikant höheren Sauerstoffpartialdruck nach 4 und 12 h einher. Wenngleich sich im weiteren Verlauf der Horovitz-Index nicht zwischen Bauch- und konservativer Lagerung unterschied, war die Rate an transplantierten Lungen nach der Bauchlagerung mit 45 gegenüber 24 % ohne deren Anwendung signifikant erhöht [39].

Eine Kohortenstudie mit 711 potenziell lungenspendenden Personen innerhalb der US-amerikanischen Texas Organ Sharing Alliance untersuchte die Einführung eines Protokolls, das vordergründig ein *Rekrutierungsmanöver* mit einer Steigerung des PEEP auf 15 cm H_2_O und des inspiratorischen Drucks auf 25 cm H_2_O für 2 h im Rahmen einer druckkontrollierten Beatmung beinhaltete. Angewendet bei einem Horovitz-Index < 300, zeigte sich ein mittlerer Zuwachs des Index um 90, einhergehend mit einer signifikanten Zunahme der transplantierten Lungen von 19 auf 37 %. In dieser Studie kamen als zusätzliche Maßnahmen Bronchoskopien mit bronchoalveolären Lavagen bei radiologisch gesicherten Infiltraten oder Zeichen der Aspiration sowie ein restriktives Volumenmanagement mit dem Ziel einer neutralen bzw. negativen Volumenbilanz zur Anwendung [35].

Der Effekt der *Bronchoskopie* dürfte im Wesentlichen auf der Beseitigung von Sekretverlegungen und der Wiedereröffnung von Atelektasen sowie der Möglichkeit der Erregerdiagnostik bei Infektionen beruhen [34, 40]. Durch die bronchoskopiegestützte Erregerdiagnostik und damit gezielte Behandlungsmöglichkeit von Infektionen scheinen sich Vorteile hinsichtlich der Transplantierbarkeit von Lungen, aber auch für die Organempfangenden zu ergeben, da die Transmission von Infektionen im besten Fall sogar vermieden werden kann [41, 42].

Ein umfangreicheres *Maßnahmenbündel* wurde in einer US-amerikanischen Studie mit 284 potenziell Organspendenden untersucht, bei denen die Zeiträume vor und nach der Einführung eines speziellen Protokolls verglichen wurden. Dieses umfasste eine lungenprotektive Beatmung mit einem inspiratorischen Spitzendruck < 30 mm Hg, einem PEEP von 8–10 cm H_2_O und Tidalvolumina von 6–8 ml/kgKG. Darüber hinaus beinhaltete das Protokoll Lagerungen mit einem 2‑stündigen Intervall, Rekrutierungsmanöver mit einem PEEP von 15 cm H_2_O für 2 h bei einem Horovitz-Index < 300, Inhalationen mit Albuterol alle 2–4 h, wiederholte Bronchoskopien in Abständen von 6–8 h, ein erweitertes hämodynamisches Monitoring zur Untersuchung des extravaskulären Lungenwassers sowie i.v.-Gaben von Hydrokortison und dem Schilddrüsenhormon Thyroxin. Im Zusammenhang mit diesem Maßnahmenbündel war ein signifikanter Anstieg der transplantierten Lungen von 19,8 auf 33,9 % zu verzeichnen [43].

Die referierten Studien liefern starke Indizien dafür, dass durch eine konsequente lungenprotektive Beatmung, wie diese auch in der aktuell geltenden, allgemeinen Leitlinie zur invasiven Beatmung empfohlen wird [17], und insbesondere die Bündelung mit Maßnahmen, die bei eingeschränkter Oxygenierung auf die Rekrutierung von Alveolen abzielen, eine deutliche *Verbesserung der Lungenfunktion im Kontext des IHA* möglich ist und hierdurch die Chance einer Transplantierbarkeit erhöht werden kann. Die Anwendung entsprechender Protokolle ist daher bereits in den nationalen und internationalen Empfehlungen enthalten (z. B. [7, 10, 12]).

Im Rahmen der organprotektiven Intensivtherapie bzw. intensivmedizinischer Maßnahmen nach der IHA-Feststellung kann es jedoch auch zu einer anhaltenden Verschlechterung der Lungenfunktion oder einem *Acute Respiratory Distress Syndrome* kommen [38, 44]. Die Spende der Lungen wird in dieser Situation unwahrscheinlich, sodass ein Verlassen der lungenprotektiven Beatmungsstrategie dann rational erscheint, wenn hierdurch eine adäquate Oxygenierung und damit die Aufrechterhaltung anderer Organfunktionen sichergestellt werden können. Auch der Einsatz extrakorporaler Unterstützungssysteme kann im Einzelfall erwogen werden, wofür bereits Anwendungserfahrungen existieren [45].

### Endokrine Störungen

Eine schwere zerebrale Schädigung mit raumfordernder Entwicklung und letztlich Übergang in den IHA geht mit der Beeinträchtigung des Hypothalamus bzw. der Hypophyse einher, was zahlreiche endokrine Konsequenzen hat. Hierzu zählt eine reduzierte oder gar vollständig erloschene Ausschüttung des thyreoidstimulierenden Hormons mit sich anschließender Reduktion des zirkulierenden Trijodthyronins und Tetrajodthyronins [20, 46]. Darüber hinaus geht der IHA mit einem Abfall der Insulinspiegels einher, verbunden mit einem intrazellulären Glucose- und somit Energiemangel sowie der Gefahr der Entwicklung einer anaeroben metabolischen Situation [20]. Vor diesem Hintergrund beinhalteten theoretische Überlegungen zum optimalen organprotektiven Management sowohl die Substitution von Schilddrüsenhormonen als auch Insulin [47–49].

In einer umfangreichen retrospektiven Registerstudie unter Einbeziehung von 63.593 Fällen aus der Datenbank des US-amerikanischen UNOS wurden die Effekte der *Substitution von Schilddrüsenhormonen* untersucht. Bei 48,7 % der registrierten Organspendenden wurden Schilddrüsenhormone (Trijodthyronin und/oder L‑Thyroxin), teils in Kombination mit anderen Hormonen (antidiuretisches Hormon, Insulin und Glukokortikoide), verabreicht, was im Vergleich zu Spendenden, die keine Substitution erhielten, mit 3,35 vs. 2,97 Organen und damit einer signifikant höheren Zahl an vermittelten Organen pro Spende assoziiert war. Erkennbar wurde ein 12,8 %iger Anstieg im Zusammenhang mit der Substitution von Schilddrüsenhormonen. Ein Gruppenunterschied zugunsten der Hormonsubstitution blieb auch dann bestehen, wenn Spendende mit ausschließlich erhaltenen Schilddrüsenhormonen gegenüber denjenigen ohne Substitution gestellt wurden. Hinsichtlich der vermittelten Organe zeichneten sich im Zusammenhang mit der Gabe von Schilddrüsenhormonen Vorteile für das Herz, die Lungen, die Nieren, das Pankreas und den Darm, aber nicht die Leber ab [50]. Warum sich der Effekt einer größeren Zahl an vermittelten Organen nicht auf die Leber übertrug, konnte nicht vollständig geklärt werden. Diese Beobachtung ist jedoch nicht konsistent, da eine weiterführende Untersuchung unter Anwendung der gleichen Datenbank im Zusammenhang mit der Gabe von Schilddrüsenhormonen bei Leberspendenden eine signifikant bessere Organfunktion nach der Transplantation im Zeitraum von 12 Monaten ergab [51]. Dass der beobachtete Effekte einer Substitution von Schilddrüsenhormonen nicht zweifelsfrei zu vereinheitlichen ist, zeigt eine Registerstudie unter Nutzung der Datenbank der International Society for Heart and Lung Transplantation mit 23.002 Fällen, die eine Herztransplantation erhielten. Etwa 69 % der Organe stammten von Spendenden, die eine Substitution von Schilddrüsenhormonen erhalten hatten. Im Ergebnis war die Substitution bei Spendenden mit einem erhöhten Risiko für ein Organversagen innerhalb von 48 h nach der Transplantation assoziiert, wohingegen sich das Langzeitüberleben nicht unterschied [52]. Zu den Limitationen der Studie gehören nichtverfügbare Kontextfaktoren wie die hämodynamische Situation zum Zeitpunkt der Substitution und fehlende Informationen hinsichtlich der eingesetzten Schilddrüsenhormone und ggf. noch weiterer Hormone. Diese Details scheinen aber von Bedeutung zu sein, da eine ungarische, retrospektive Kohortenstudie mit 297 transplantierten Herzen die Beobachtung lieferte, dass die Substitution von L‑Thyroxin, die protokollgemäß als eine Option bei hämodynamischer Instabilität der Spendenden, teils in Kombination mit Methylprednisolon, Desmopressin bzw. Vasopressin, zum Einsatz kam, mit einer geringeren Rate an Organdysfunktionen und einer geringeren Sterblichkeit innerhalb von 2 Jahren nach der Transplantation assoziiert war [53].

Dagegen erscheint die Wahl des Schilddrüsenhormons (*Trijodthyronin vs. L‑Thyroxin*) zumindest in Bezug auf hämodynamische Parameter während der organprotektiven Therapie bzw. Maßnahmen und die Anzahl an realisierten Spenden von untergeordneter Bedeutung, wie eine randomisierte Studie mit 37 potenziell Organspendenden aus den USA ergab [54]. Bezogen auf die Versorgungspraxis ergab eine US-amerikanische Umfrage hinsichtlich des gewählten Zeitpunkts der Substitution von Schilddrüsenhormonen insbesondere in Bezug auf hämodynamische Kriterien und der angewandten Dosis deutliche Variationen, was die bestehenden Unsicherheiten verdeutlicht [55].

Für eine bestmögliche Bewertung *kausaler Effekte der Schilddrüsenhormonsubstitution* sind daher randomisierte Studien besonders wichtig. Eine US-amerikanische, randomisierte Pilotstudie schloss potenziell Herzspendende ein, bei denen ein Weaning der Vasopressoren im Zuge der Volumentherapie und Gabe von Methylprednisolon gelungen war und echokardiographisch eine linksventrikuläre Ejektionsfraktion < 60 % vorlag, wobei 17 Fälle L‑Thyroxin erhielten (20 μg i.v. als Bolus, nachfolgend 10 μg/h für 8 h) und 11 keine Substitution. Im Ergebnis unterschied sich der nach 8 h erkennbare Anstieg der linksventrikulären Ejektionsfraktion nicht signifikant zwischen den beiden Gruppen, wenngleich sich mit 59 vs. 27 % ein Trend zu einer höheren Rate an realisierten Spenden im Zusammenhang mit der Hormonsubstitution zeigte [56]. In einer deutlich umfangreicheren randomisierten Studie unter Einbeziehung von 15 US-amerikanischen Organisationen, die mit der Organspende bzw. -transplantation befasst sind, und 852 potenziell Herzspendenden mit bestehender hämodynamischer Instabilität konnte dieser Trend jedoch nicht bestätigt werden. Im Zusammenhang mit der Gabe von L‑Thyroxin (30 μg/h i.v. für 12 h) zeigte sich mit 54,9 vs. 53,2 % kein Unterschied hinsichtlich der realisierten Spenden. Auch fanden sich keine Auswirkungen auf die Vasopressorgabe und die echokardiographisch beurteilte Ejektionsfraktion, wohingegen sich im Zuge der Substitution von L‑Thyroxin vermehrt Hypertensionen und Tachykardien, die zu einer vorzeitigen Beendigung der Infusion führten, zeigten [57].

Insgesamt liegt *keine überzeugende Evidenz für die Substitution von Schilddrüsenhormonen* während der organprotektiven Intensivtherapie bzw. intensivmedizinischer Maßnahmen nach der IHA-Feststellung vor. Folglich ist diese beispielsweise in kanadischen und US-amerikanischen Empfehlungen auch nicht enthalten [7, 10]. In Deutschland existiert in abgeschwächter Form die Empfehlung, dass eine routinemäßige Substitution nicht erfolgen soll, diese bei hämodynamischer Instabilität und einer linksventrikulären Ejektionsfraktion < 45 % jedoch erwogen werden kann [12, 29].

Die niederschwellige Anwendung von *Insulin* während der organprotektiven Therapie gehört zu den Faktoren, die besonders schwer hinsichtlich etwaiger Effekte zu beurteilen sind. Im Rahmen allgemeiner intensivmedizinischer Strategien werden auftretende Hyperglykämien oft mit Insulin behandelt, sodass randomisierte Studien hierzu kaum möglich sind. Hinzu kommt, dass die außerhalb von Deutschland vielerorts genutzten Protokolle neben der Insulin- auch die Glukokortikoidgabe mit anzunehmendem negativem Effekt auf den Glucosestoffwechsel beinhalten, sodass die Ableitung von Erkenntnissen allein aus Registerstudien erschwert ist [58]. Unter Nutzung der US-amerikanischen Datenbänke des OPTN und des UNOS zeigte sich bei insgesamt 12.841 Personen mit erfolgter Pankreastransplantation die Überlebensrate in einem 10-jährigen Beobachtungszeitraum nicht davon abhängig, ob die Spendenden Insulin erhielten oder nicht. Als Ausdruck einer hohen methodischen Qualität wurden Organempfangende, die nicht an einem typischen (d. h. Typ-1- oder Typ-2‑)Diabetes mellitus litten oder ein Organ von einer an Diabetes mellitus erkrankten Person erhielten, nicht berücksichtigt [59]. Eine Registerstudie aus dem Vereinigten Königreich ergab unter Einbeziehung der UK Transplant Registry mit 2168 Pankreastransplantationen dagegen die Beobachtung, dass eine Insulintherapie bei Spendenden mit einer höheren Wahrscheinlichkeit für ein Organversagen innerhalb von 3 Monaten nach der Transplantation assoziiert war [60].

Eine Empfehlung zur niederschwelligen Insulingabe kann daher aus den vorgenannten Studien nicht abgeleitet werden. Vielmehr sollten *im intensivmedizinischen Kontext übliche Strategien zur Vermeidung einer relevanten Hyperglykämie* Anwendung finden. Im Kontext des IHA kann die Insulingabe zudem eine Bedeutung bei der Herstellung der Voraussetzungen für die richtliniengemäßen Feststellung zukommen, indem eine relevante Hyperglykämie als metabolische Störung beseitigt wird.

### Inflammation und Infektion

Während der organprotektiven Therapie und insbesondere im zeitlichen Zusammenhang mit dem IHA kann es zu einer systemischen Inflammationsreaktion kommen, welche durch die Erkrankung, die der zerebralen Schädigung zugrunde liegt, verstärkt werden kann [61]. Hinzu kommen infektiologische Komplikationen im intensivmedizinischen Verlauf wie aspirations- und beatmungsassoziierte Pneumonien. Die potenzielle Kombination aus unspezifischer Inflammationsreaktion und relevanter Infektion mit einer Beeinträchtigung beispielsweise der Lungenfunktion macht das immunmodulierende und antiinfektive Management bei potenziell Organspendenden zu einer besonderen Herausforderung.

Allgemein angenommen werden eine den IHA begleitende Ausschüttung proinflammatorischer Zytokine sowie eine *systemische Immunreaktion* [20, 61]. Erhöhte Serumkonzentrationen wurden in mehreren Untersuchungen beispielsweise für Interleukin‑1 und -6 sowie den Tumor-Nekrose-Faktor‑α, das C‑reaktive Protein und Prokalzitonin bei Herz- bzw. Lungenspendenden nachgewiesen (z. B. [62, 63]). Eine aktuelle Arbeit, bei der Serumproben von 27 Spendenden aus der Quality-in-Organ-Donation-Biobank des Vereinigten Königreichs ausgewertet wurden, lieferte jedoch keinen Anstieg des Interleukin‑6 und Tumor-Nekrose-Faktors‑α im Nachgang der IHA-Feststellung während eines Verlaufs bis zu 30 h [64]. Andere Einflüsse und hierbei v. a. Anteile des intensivmedizinischen Verlaufs vor der Feststellung des IHA scheinen demnach einen relevanten Einfluss auf den beobachtenden Anstieg proinflammatorischer Marker zu haben.

Im Zuge des IHA wird neben der systemischen Inflammationsreaktion jedoch auch ein *reduzierter systemischer Kortisolspiegel* diskutiert [20]. Diese Annahme stützt eine Kohortenstudie aus Griechenland mit insgesamt 37 Fällen, die eine schwere zerebrale Schädigung aufwiesen, und von denen 17 einen IHA entwickelten. Bei Personen mit festgestelltem IHA wurden im Vergleich zu denjenigen ohne IHA signifikant reduzierte Kortisolspiegel festgestellt. Auch die Stimulation mit adrenokortikotropem Hormon führte bei Personen mit IHA zu einem verminderten Anstieg der Kortisolwerte [65]. Vor dem Hintergrund des anzunehmenden Nebeneinanders von systemischer Immunreaktion und vermindertem Kortisolspiegel wurde die Gabe von Glukokortikoiden mit möglichen positiven Effekten während der organprotektiven Therapie bzw. Maßnahmen und auf die Organfunktion nach einer Transplantation diskutiert. Mit Beteiligung der Deutschen Stiftung Organtransplantation untersuchte eine umfangreiche Studie mit 100 Organspendenden unter Anwendung eines randomisierten Designs die Gabe von *Methylprednisolon*. In 50 Fällen wurde Methylprednisolon mit einer Einmalgabe von 250 mg i.v. und nachfolgend 100 mg/h ab dem Zeitpunkt der Zustimmung zur Organspende eingesetzt. Im Ergebnis war die Methylprednisolongabe mit höheren Serumspiegeln von Steroiden und geringeren Werten von Interleukin‑2 und -6 sowie dem Tumor-Nekrose-Faktor‑α und anderen proinflammatorischen Zytokinen assoziiert. Bezogen auf die Anzahl der realisierten Lebertransplantationen, ging die Methylprednisolongabe mit 22 vs. 36 % und damit einer geringeren Rate an mikroskopisch gesicherten Abstoßungsreaktionen innerhalb eines 6‑monatigen Beobachtungszeitraums einher [66]. Eine Reduktion proinflammatorischer Zytokine infolge der Methylprednisolongabe nach dem vorgenannten Schema wurde in einer weiteren deutschen Studie mit insgesamt 53 Fällen beobachtet. Zusätzlich zu Serumproben, die nach der Feststellung des IHA gewonnen wurden, erfolgten auch Zytokinanalysen in Leberbiopsien, die unmittelbar nach der Laparotomie während der Explantation gewonnen wurden. Hierdurch konnte das proinflammatorische Milieu direkt im gespendeten Organ beurteilt werden [63]. Den Effekt von Methylprednisolon auf andere Organe untersuchte eine randomisierte Studie aus dem Vereinigten Königreich mit 182 potenziell Lungenspendenden. Von diesen erhielten 15 Methylprednisolon (1 g i.v. als Einmalgabe) und 14 eine Kombination mit zusätzlich Trijodthyronin (0,8 μg/kgKG i.v. als Bolus, nachfolgend 0,113 μg/kgKG und h), was u. a. mit einer geringeren Ausprägung des extravaskulären Lungenwassers assoziiert war, jedoch nicht mit einer erhöhten Zahl an für eine Spende geeigneten Lungen einherging [67]. Mit dem gleichen Dosierungsschema untersuchte eine randomisierte Studie aus dem Vereinigten Königreich 80 potenziell Herzspendende, von denen 19 Methylprednisolon und 20 eine Kombination mit zusätzlich Trijodthyronin erhielten. Erkennbar wurde eine Verbesserung des Herzindex in der gesamten Stichprobe, was vordergründig auf begleitende Maßnahmen zur Optimierung der kardialen Auswurfleistung zurückzuführen war. Die Veränderungen des Herzindex und die Rate an für eine Spende geeigneten Herzen waren jedoch nicht durch die untersuchten Interventionen moduliert [68].

Die *kombinierte Gabe von Methylprednisolon und Trijodthyronin* war auch Gegenstand deutlich umfangreicherer Untersuchungen, darunter eine retrospektive Studie auf der Basis der Datenbank des US-amerikanischen UNOS mit 10.292 Fällen. Als Teil eines Maßnahmenbündels zusammen mit der Anwendung von Vasopressin sowie Trijodthyronin/L-Thyroxin war die Methylprednisolongabe mit einer 22,5 %igen Zunahme der pro Spende realisierten Organe assoziiert. Die größten Zuwächse in der Wahrscheinlichkeit für die Transplantation einzelner Organe zeigten sich dabei mit 7,3 % für die Nieren und mit 6 % für das Pankreas [69].

Hinsichtlich der optimalen *Dosis bzw. Wahl des Glukokortikoids* bestehen deutliche Unsicherheiten. So lieferte eine US-amerikanische Studie mit 132 potenziell Lungen- bzw. Herzspendenden keinen Unterschied in der Rate der transplantierten Organe im Vergleich von Methylprednisolon in einer Dosierung von 15 mg/kgKG (mit Wiederholung alle 24 h) und Hydrokortison mit 300 mg (gefolgt von 100 mg alle 8 h) [70]. Die angenommene Hypothese, dass die Gabe von Glukokortikoiden einen supprimierenden Einfluss auf erhöhte proinflammatorische Zytokine mit hieraus erwachsenden Vorteilen in Bezug auf die Transplantierbarkeit von Organen hat, scheint zumindest teilweise durch die vorgenannten Studien gestützt. Daneben existiert aber auch die in einer Studie mit 79 potenziell Organspendenden aus dem Vereinigten Königreich gemachte Beobachtung, dass die Anwendung von Methylprednisolon (1 g i.v. als Einmalgabe) einzeln oder in Kombination mit Trijodthyronin (0,8 μg/kgKG i.v. als Bolus, nachfolgend 0,113 μg/kgKG und h) nicht zu einer Veränderung pro-/inflammatorischer Zytokine und Marker wie Interleukin‑1 und -6 sowie dem Tumor-Nekrose-Faktor‑α führte [62].

Möglicherweise spielt ein *kreislaufstabilisierender Effekt* eine relevantere Rolle, wofür eine prospektive kontrollierte Studie aus Frankreich spricht, in der die Gabe von Hydrokortison mit einer geringeren Dosis von Norepinephrin und einer kürzeren Anwendungsdauer von diesem assoziiert war [71]. Wenngleich der Hintergrund der beobachteten Effekte nicht vollständig geklärt ist, *enthalten nationale und internationale Handlungsempfehlungen die Gabe von Glukokortikoiden,* teilweise in Abhängigkeit von der hämodynamischen Situation [7, 8, 10–12, 29, 72, 73]. Hierbei sollte berücksichtigt werden, dass die Studienlage für diese Empfehlung nicht besonders überzeugend ist [74]. Gerade der Effekt im Falle einer vorliegenden stabilen hämodynamischen Situation muss kritisch hinterfragt werden. Derzeit ungeklärt sind daher der optimale Zeitpunkt des Beginns einer Glukokortikoidgabe und die optimale Dosierung. Vor dem Hintergrund pathophysiologischer Überlegungen mit anzunehmender Ausschüttung proinflammatorischer Zytokine bei schweren zerebralen Schädigungen und einem unmittelbar bevorstehenden IHA würde sich eine Rationale für einen frühzeitigen Beginn ergeben; diese sollte jedoch zunächst in Studien überprüft werden.

In der einschlägigen Literatur beziehen sich konkrete Empfehlungen für die *Behandlung von Infektionen* im Zuge der organprotektiven Therapie hauptsächlich auf Erkrankungen der Spendenden wie HIV, Hepatitis B und C mit der resultierenden Frage, ob Organe in diesen Fällen transplantiert werden können [10, 75, 76]. Eine systematische Untersuchung von Infektionen wie aspirations- bzw. ventilatorassoziierten Pneumonien während der organprotektiven Therapie vor und intensivmedizinischer Maßnahmen nach der Feststellung des IHA existiert bislang nicht. Dass Infektionen im zeitlichen Zusammenhang mit dem IHA von Bedeutung sind, lassen mehrere Untersuchungen erkennen. Beispielsweise ergab eine datenbankbasierte Studie aus der Türkei mit insgesamt 102 potenziell Spendenden eine Häufigkeit von ca. 24 %, wobei Blutstrominfektionen mit ca. 42 % und Infektionen des Respirationstrakts mit ca. 38 % die häufigsten Formen waren [77]. Da in der Literatur Infektionen für gemischte intensivmedizinische Kollektive in bis zu 60 % der Fälle beschrieben werden [78], erscheint die Rate an Infektionen bei potenziell Spendenden nicht erhöht. Dem steht die Beobachtung gegenüber, dass in einer retrospektiven US-amerikanischen Studie mit 440 Spendenden in 97 % der Fälle ein Antibiotikum eingesetzt wurde und es sich bei 71 % um ein Breitspektrumantibiotikum handelte [79]. Allgemein erhöhen Antibiotika das Risiko für die Entwicklung multiresistenter Erreger, wofür es bei Organspendenden klare Hinweise gibt, und woraus sich Konsequenzen für die Organempfangenden ergeben könnten [80].

Erschwert wird die Indikationsstellung einer antiinfektiven Behandlung während der organprotektiven Therapie bzw. Maßnahmen dadurch, dass bei potenziell Organspendenden *Inflammationsmarker* wie das C‑reaktive Protein, Interleukin‑6 und Prokalzitonin regelhaft erhöht sind [62]. Eine aus Frankreich stammende, retrospektive Kohortenstudie mit 82 potenziell Organspendenden bestätigte dies insofern, dass ein erhöhtes Prokalzitonin in 46 % der Fälle vorlag und sich dies nicht zwischen Verläufen mit und ohne Infektion unterschied [81]. Daher eignen sich Inflammationsparameter kaum für die antiinfektive Therapiesteuerung im Kontext des IHA.

Aufgrund dieser Beobachtungen und der schlechten Datenlage wird das antiinfektive Management in internationalen Empfehlungen kaum berücksichtigt und in Anlehnung an allgeneine intensivmedizinische Prinzipien eine *Antibiotikagabe nur bei nachgewiesener Infektion, Sepsis bzw. Bakteriämie empfohlen*, im Falle einer Darmspende auch teilweise prophylaktisch [7, 8, 10, 72, 73, 76]. Rational erscheinen hierbei die regelmäßige Reevaluation des Antibiotikums und der Behandlungsdauer sowie die Einbeziehung der vielerorts bereits etablierten Antibiotic-Stewardship-Teams.

### Medikamentöse Maßnahmen, die auf die Organprotektion nach der Entnahme abzielen

Für *Dopamin* wurden v. a. im Zusammenhang mit der Transplantation von Nieren antioxidative Eigenschaften, die während der Kälte-Ischämie-Phase oxidativen Stress mindern und so potenzielle Schäden reduzieren könnten, diskutiert [82–85]. Eine randomisierte Studie unter Beteiligung von 60 europäischen Zentren ergab bei der Auswertung 487 transplantierter Nieren eine reduzierte Dialysepflicht und verbesserte Frühfunktion bei vorheriger Dopamingabe [82]. Zudem zeigte eine spätere Analyse derselben Kohorte einen positiven Effekt auf das 5‑Jahres-Transplantatüberleben, wenn Dopamin über mindestens 6 h appliziert wurde [86]. Die Deutsche Transplantationsgesellschaft (DTG) empfahl im Jahr 2017 auf Grundlage der bis dahin verfügbaren Evidenz eine routinemäßige Gabe von niedrigdosiertem Dopamin (4 µg/kgKG und min) bei Organspendenden unmittelbar nach Feststellung des IHA bis zur Organentnahme [87].

Neuere Daten werfen jedoch die Frage auf, ob die Empfehlung zur generellen Anwendung von Dopamin im Kontext organprotektiver intensivmedizinischer Maßnahmen aufrechterhalten werden kann. Hintergrund ist eine Metaanalyse, die 7 randomisierte Studien mit insgesamt 767 Fällen umfasste und keinen konsistenten Vorteil einer niedrigdosierten Dopamingabe ergab. Während eine signifikante Steigerung der Urinausscheidung im Nachgang der Transplantation beobachtet wurde, blieb der *Einfluss von Dopamin auf das Überleben transplantierter Nieren ohne statistische Signifikanz*. Auch hinsichtlich der Dialysepflicht nach Transplantation waren die Ergebnisse insofern uneinheitlich, dass die in früheren Einzelstudien gemachte Beobachtung einer Reduktion in der Gesamtanalyse nicht bestätigt werden konnte. Ebenso zeigte sich keine signifikante Verbesserung der frühen Transplantatfunktion, gemessen am Serum-Kreatinin-Spiegel [88].

Bezogen auf die *Funktion von Organen abseits der Nieren* existieren Studien, die auf transplantierte Lungen und Herzen im Zusammenhang mit der Gabe von Dopamin fokussierten. Eine prospektive Beobachtungsstudie aus den USA mit 6052 hirntoten Organspendern, von denen 1811 Lungen transplantiert wurden, identifizierte die Gabe von Dopamin im Spendermanagement als unabhängigen negativen Prädiktor für das Transplantatüberleben der Lunge (Odds Ratio 0,19; *p* = 0,01) [38]. Eine Substudie der vorgenannten randomisierten Studie zum Einfluss von Dopamin auf die Funktion transplantierter Nieren [82] berücksichtigte Fälle, die auch eine Herzspende aufwiesen. Es resultierte eine Kohorte mit 93 Herztransplantationen, in der die Anwendung von Dopamin mit einer verbesserten Überlebensrate innerhalb von 3 Jahren nach der Transplantation einherging, wohingegen sich kein Effekt auf die linksventrikuläre Pumpfunktion, die Notwendigkeit linksventrikulärer Unterstützungssysteme und die Rate an bioptisch gesicherten Abstoßungsreaktionen zeigte [89].

Insbesondere vor dem Hintergrund der neueren Erkenntnisse in Bezug auf das Überleben transplantierter Nieren und deren Funktion haben mehrere Länder die Empfehlungen hinsichtlich der Anwendung von Dopamin bereits überarbeitet. Beispielsweise wurde in Kanada bereits im Jahr 2020 von der Dopamingabe abgeraten [7]. Diese Empfehlung wurde in der Aktualisierung der Leitlinie im Jahr 2024 erneut bestätigt, wobei als Gründe ein erhöhtes Risiko für Herzrhythmusstörungen sowie eine mögliche Erhöhung der Mortalität bei kritisch Kranken angeführt wurden [72, 90]. Vor diesem Hintergrund erscheint eine Überprüfung der in Deutschland bestehenden DTG-Empfehlung sinnvoll, um potenziell nachteilige Effekte der Dopamingabe auf das Transplantatüberleben zu berücksichtigen.

## Schlussfolgerung

Pathophysiologische Besonderheiten im zeitlichen Zusammenhang mit dem IHA führen zu Herausforderungen während der organprotektiven Intensivtherapie vor bzw. der intensivmedizinischen Maßnahmen nach der IHA-Feststellung. Für einzelne Aspekte wie den Diabetes insipidus, eine unerwartet diagnostizierte linksventrikuläre Dysfunktion, eine eingeschränkte Lungenfunktion sowie endokrine Störungen und das Auftreten proinflammatorischer Zytokine existieren zahlreiche Studien, v. a. auf der Basis von Behandlungskohorten und Registern. Diese deuten darauf hin, dass die i.v.-Gabe von Vasopressin und Glukokortikoiden sowie die konsequente lungenprotektive Beatmung mit ggf. Rekrutierungsmanövern zumindest in bestimmten Situationen mit einer Verbesserung der Hämodynamik und Lungenfunktion sowie einer besseren Allokationsrate der Spenderorgane einhergehen.

Aus praktischer Sicht sind jedoch Detailfragen wie beispielsweise der optimale Zeitpunkt der Anwendung von Vasopressin und Glukokortikoiden innerhalb des Kontinuums vom unmittelbar bevorstehenden zum festgestellten IHA sowie der sich anschließenden Phase unbeantwortet. Unklar ist auch, welche intensivmedizinischen Konsequenzen sich aus der Beobachtung ableiten lassen, dass eine linksventrikuläre Dysfunktion bei zuvor herzgesunden Spendern im Verlauf eine Erholung zeigen kann – und ob sich daraus eine veränderte Einschätzung der Transplantierbarkeit des Herzens ergibt. Daneben existieren anhaltend kontroverse Diskussionen hinsichtlich der Substitution von Schilddrüsenhormonen und der niederschwelligen Insulingabe. Gleiches gilt für die Applikation von Dopamin als organprotektive Maßnahme, die auf den Zeitraum nach der Organentnahme abzielt. Insbesondere vor dem Hintergrund neuerer Daten erscheint eine kritische Neubewertung der bisher in Deutschland vorhandenen Empfehlung für die Dopamingabe nach der IHA-Feststellung sinnvoll.

Allgemein ist die Evidenz für spezielle organprotektive bzw. organfunktionserhaltende Maßnahmen vergleichsweise gering. Zu den Gründen gehört eine erschwerte kausale Beurteilung der beobachteten Effekte, da in den entsprechenden Studien ein randomisiertes Studiendesign selten zur Anwendung kam und die genutzten Protokolle zumeist Maßnahmenbündel enthielten. Forschungsinitiativen mit differenzierten Fragestellungen im zeitlichen Zusammenhang mit dem IHA und den Inhalten der organprotektiven Intensivtherapie bzw. intensivmedizinischen Maßnahmen, die auf eine Aufrechterhaltung der Organfunktion abzielen, sind daher unerlässlich. Insbesondere für die Beurteilung kausaler Effekte einzelner Maßnahmen wären randomisierte kontrollierte Studien mit ausreichenden Fallzahlen hilfreich. Daneben würden Registerstudien die Möglichkeit bieten, Versorgungsaspekte zu erkunden und mithilfe von Subgruppenanalysen Kohorten zu identifizieren, die von speziellen Maßnahmen profitieren könnten. Mit den US-amerikanischen Registern OPTN und UNOS existieren Vorbilder, die Impulse für den weiteren Ausbau nationaler, vernetzter Forschungsstrukturen in Deutschland geben könnten, insbesondere unter Einbeziehung des bereits bestehenden Deutschen Nationalen Transplantationsregisters [91]. Derartige Studien würden eine gute Grundlage für die Erstellung einer evidenzbasierten Leitlinie auch im deutschsprachigen Raum liefern.

## Fazit für die Praxis

- Intensivmedizinische Maßnahmen bei Personen mit potenzieller Organspende orientieren sich im Wesentlichen an den Prinzipien der evidenzbasierten Intensivmedizin, insbesondere im Hinblick auf die Beatmung sowie Volumen- und Katecholamintherapie.
- Vasopressin zeigte in Studien sowohl hinsichtlich der Kreislaufstabilisierung als auch bei der frühzeitigen Behandlung des zentralen Diabetes insipidus potenzielle Vorteile und kann daher niederschwellig erwogen werden.
- Der Einsatz von Glukokortikoiden gemäß gängigen Dosierungsstandards lieferte in Studien positive Effekte insbesondere hinsichtlich der Kreislaufstabilisierung, sodass der niederschwellige Einsatz unter Berücksichtigung potenzieller Nebenwirkung erwogen werden kann.
- Eine unkritische, routinemäßige Gabe von Dopamin sollte aufgrund der aktuellen Datenlage zumindest so lange vermieden werden, bis weiterführende Untersuchungen zum Sicherheitsprofil vorliegen.
- Es besteht ein Bedarf an klinischen Studien, die speziell auf die Phase des unmittelbar bevorstehenden sowie eingetretenen IHA ausgerichtet sind, um Evidenz für einzelne Aspekte wie den optimalen Zeitpunkt intensivmedizinischer Maßnahmen zu generieren – insbesondere im Hinblick auf das endokrine System und infektiologische Fragestellungen.
